# Neurofilament Light Chain Concentration in the Prediction of Treatment Response in Multiple Sclerosis

**DOI:** 10.1111/ene.70505

**Published:** 2026-02-02

**Authors:** Nahid Moradi, Sifat Sharmin, Charles B. Malpas, Jens Kuhle, Pascal Benkert, David Leppert, Eva Kubala Havrdová, Dana Horáková, Pavlína Kleinová, Tomas Uher, Ali Manouchehrinia, Jan Hillert, Tomas Olsson, Ingrid Kochum, Bruce V. Taylor, Michael Barnett, Trevor J. Kilpatrick, Katherine Buzzard, Tomas Kalincik

**Affiliations:** ^1^ Core, Department of Medicine The University of Melbourne Melbourne Victoria Australia; ^2^ Neuroimmunology Centre, Department of Neurology The Royal Melbourne Hospital Melbourne Victoria Australia; ^3^ Melbourne School of Psychological Sciences, the University of Melbourne Australia; ^4^ Neurologic Clinic and Policlinic, MS Centre and Research Centre for Clinical Neuroimmunology and Neuroscience Basel, University of Basel Basel Switzerland; ^5^ Clinical Trial Unit, Department of Clinical Research University Hospital Basel, University of Basel Basel Switzerland; ^6^ Department of Neurology and Center of Clinical Neuroscience First Faculty of Medicine, Charles University and General University Hospital Prague Czech Republic; ^7^ Department of Clinical Neuroscience Karolinska Institutet Stockholm Sweden; ^8^ Centre for Molecular Medicine, Karolinska University Hospital Stockholm Sweden; ^9^ Menzies Institute for Medical Research, University of Tasmania Hobart Tasmania Australia; ^10^ Brain and Mind Centre, the University of Sydney Sydney New South Wales Australia; ^11^ Sydney Neuroimaging Analysis Centre Camperdown New South Wales Australia; ^12^ The Florey Institute of Neuroscience and Mental Health, University of Melbourne Parkville Victoria Australia; ^13^ Department of Neurology Box Hill Hospital Melbourne Australia

**Keywords:** multiple sclerosis, neurofilament light, prediction, principal component analysis, treatment response

## Abstract

**Introduction:**

Management of multiple sclerosis (MS) revolves around timely initiation of effective disease‐modifying therapy. Here we investigate the additive predictive value of age‐adjusted normalised neurofilament light chain (NfL) concentrations when combined with a clinicodemographic model of treatment response.

**Methods:**

Data were obtained from three sources: the University Hospital Basel, the SET cohort in Prague, and EIMS and IMSE cohorts from Sweden. NfL samples were collected within 90 days of baseline, age‐adjusted and normalised using a reference population. Principal component analysis reduced the dimensionality of clinicodemographic predictors. Cox proportional hazards models estimated cumulative hazards of relapse, 6‐month confirmed disability worsening and 9‐month confirmed disability improvement, with and without NfL. Uno's concordance index compared prediction accuracy across pooled and treatment‐specific models.

**Results:**

The study included 1716 individuals across three therapies: interferon β (*n* = 554), fingolimod (*n* = 307) and natalizumab (*n* = 369). Clinicodemographic characteristics were associated with relapse and disability outcomes. While NfL showed no association in the pooled cohort, in the natalizumab group, higher NfL predicted lower probability of disability improvement (HR = 0.819, 95% CI: 0.814–0.823). Pooled models predicted outcomes with moderate accuracy (relapse: 63.4%, disability worsening: 56.4%, improvement: 67.7%), with minimal contribution from NfL. In treatment‐specific models, NfL‐inclusive accuracy ranged from 51.3%–62.2% (relapse), 54.3%–60.3% (worsening) and 65%–67.9% (improvement), closely matching models without NfL.

**Conclusion:**

In well‐characterised MS patients treated with interferon β, fingolimod or natalizumab, clinicodemographic information provides modest prognostic value; however, NfL adds minimal incremental utility.

## Graphical abstract

This graphical abstract shows that serum NfL was evaluated alongside clinicodemographic variables that were reduced using principal component analysis (PCA). These components were then incorporated into a combined prognostic model. The addition of serum NfL did not result in any significant improvement in prognostic accuracy in the pooled cohort analysis beyond the clinicodemographic data alone.

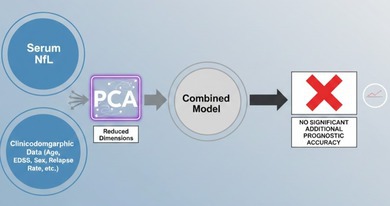

## 1 Introduction

Many presently available disease‐modifying therapies (DMTs) have been shown to reduce relapses and disability accrual in multiple sclerosis (MS). However, response to therapy can be highly variable among individuals. This variability can be associated with demographic, clinical, paraclinical and genetic characteristics [1, 2]. Neurofilament light (NfL) chain is an emerging sensitive biomarker of neuronal damage that has drawn attention for its potential role in monitoring of treatment response [3]. However, its utility as a predictive marker of response to therapy remains to be established.

NfL is a cytoskeletal protein that is particularly abundant in higher calibre myelinated axons. As a result of neuronal injury, NfL enters the interstitial fluid and the CSF and subsequently the bloodstream, albeit at a much lower concentration than in the CSF [4]. NfL is a pathophysiological biomarker of neuronal injury and thus could reflect the therapeutic effects of DMTs on disease activity [5, 6, 7]. Recently, its value as a predictor of short‐term clinical MS activity has been demonstrated [5, 8, 9, 10, 11, 12].

Predicting individual treatment response is challenging. A small number of presently available predictive models have focussed on the demographic and clinical indicators of future treatment response with varying approaches [13, 14, 15]. Our approach – the Crystal Ball prediction model, developed using the MSBase international registry [16] – has also demonstrated the feasibility of carrying out this task [15]. The original model helps predict the probability of relapse and change in disability over a horizon of 4 years within groups treated with different DMTs, conditional on individuals' demographic and clinical characteristics. This model uses principal component analysis [17] as a means of dimensionality reduction to retain relevant demographic and clinical information without risking overfitting and is able to handle data missingness. Its prediction accuracy in external validation cohorts is satisfactory for relapse and disability outcomes; however, its discriminative ability remains a limiting factor [15, 18].

In the light of the recent advances in NfL research, it is imperative to investigate the contribution of this biomarker for predicting response to specific treatments alongside the routinely considered prognostic factors. To carry out this objective, we assessed the contribution of NfL in the context of prediction models adjusted for established clinicodemographic predictors represented through the principal components (PCs). These models predict individual outcomes in response to therapies for MS in the presence of varying therapy durations which are representative of real‐world practice.

## 2 Methods

### 2.1 Study Population

Data from three sources, the University Hospital of Basel [19], the SET study from the General University Hospital in Prague [20] and EIMS [21] and IMSE [22] cohort studies represented by Karolinska Institute, were mapped and collated to identify patients with: relapsing forms of MS based on the McDonald Criteria 2005, 2010 or 2017 [23, 24, 25] and minimum dataset availability. The minimum dataset consists of information on sex, date of birth, year of first clinical presentation, disease course and treating centre, relapse dates, clinical visits, disability assessment and therapy history. Clinically isolated syndrome was re‐evaluated in keeping with the most recent McDonald criteria when CSF analysis was available [25]. Further, patient records included information on serum or EDTA‐treated plasma NfL concentration within 90 days of a disability score. The date of the disability score within this 90 days window was considered the study baseline. Except for those newly diagnosed, a baseline was preceded by at least 6 months of documented follow‐up (as indicated by a prior disability score) and followed by at least 6 months of post‐baseline follow‐up, with a minimum of 1 disability score recorded during each post‐baseline year. The included participants were either treated with a DMT prior to baseline or started therapy within 60 days of baseline and remained treated for at least 180 days thereafter.

### 2.2 Neurofilament Light Chain Assay

Blood samples were collected in accordance with the protocols of the Swiss MS Cohort, SET study and EIMS and IMSE cohorts. Serum (Basel, Prague) or EDTA‐treated plasma (Sweden) were obtained and stored at −80° [26]. To quantify the concentration of NfL, the serum obtained in Basel was analysed using a commercially available single‐molecular array (simoa) assay (Quanterix, Billerica, MA, USA) as described elsewhere [27]. The serum obtained for the SET study (Prague) was transferred to University Hospital of Basel, where it was analysed using an in‐house simoa assay as described elsewhere [11, 28]. The EDTA‐treated plasma samples obtained for the EIMS and IMSE cohorts were transferred to University Hospital of Basel, where NfL was measured using the commercial NfL simoa assay (Quanterix, Billerica, MA, USA) [28, 29]. The in‐house assay was benchmarked against the commercial assay and diligent quality control and quality assurance processes were followed. Overall, NfL results were surveyed from January 2001 to July 2020 across the study sites.

The measured concentrations of NfL were converted into *z*‐scores normalised for patients' age using a reference population of healthy controls and people with MS from the University Hospital of Basel [5, 28]. This was achieved by fitting a generalised additive model for location, scale and shape while adjusting for age and body mass index (where available) as described elsewhere [5].

### 2.3 Study Outcomes

This study modelled cumulative hazards of relapses, confirmed disability worsening and confirmed disability improvement, as these outcomes showed the greatest accuracy in the original study and its validation [15, 18, 30]. Relapses were defined as occurrence of a new or exacerbation of existing neurological signs or symptoms lasting at least 24 h, in the absence of a febrile disease and at least 30 days from a previous relapse [31]. Disability was quantified with the Expanded Disability Status Scale (EDSS) by Neurostatus‐certified raters [32]. Confirmed disability worsening (CDW) was defined as an increase in EDSS score by ≥ 1.5 steps if baseline EDSS was 0, by ≥ 1 step if baseline EDSS was 1–5.5 and by ≥ 0.5 steps if baseline EDSS was ≥ 6, confirmed by EDSS scores recorded outside of a relapse (≥ 30 days) over at least 6 subsequent months and sustained for the remainder of the recorded follow‐up [33]. Confirmed disability improvement (CDI) was defined as a decrease in EDSS by 1.5 steps if baseline EDSS was ≤ 1.5, ≥ 1 step if baseline EDSS was 2–6 and ≥ 0.5 step if baseline EDSS was ≥ 6.5, confirmed over a period of at least 9 months and sustained for the remainder of the recorded follow‐up.

### 2.4 Statistical Analyses

First, we investigated the contribution of NfL *z* scores at baseline to a general prognostic model of MS relapses and disability in a pooled cohort (adjusted for DMT treatment duration and exposure). Second, we evaluated the contribution of baseline NfL concentration to the prediction of treatment response for DMTs with enough patients and events (≥ 150 eligible patients).

The Crystal Ball models use hazard functions to model cumulative hazards of relapses, CDW and CDI, conditional on the principal components (PCs) derived from clinical and demographic characteristics (to reduce the dimensionality of the large dataset). We used the original PC matrix that was developed using the 2015 MSBase data extract [15, 16]. PC1 mainly summarises overall neurological disability (EDSS) and its facets and the history of previous disease‐modifying therapy and therapy response. PC2 mainly represents relapses – their frequency, severity and recovery as well as patient age and MS course. PC3 mainly represents the phenotype of MS symptoms at onset. Using the variable loadings from the original study, we calculated the three principal components for each of the participants included in the present study.

Cox proportional hazards models were used to model the cumulative hazards of first relapse event, CDW and CDI after baseline with the application of generalised estimating equations for clustering by study site. The models of MS prognosis evaluated the associations between NfL and the three outcomes, adjusted for the three principal components (which summarise patients' characteristics, including clinical history, disability, recent disease activity and other information), treatment duration at baseline and therapy category at baseline.

The models of treatment‐specific disease activity evaluated the association between NfL and the three outcomes, adjusted for the three principal components and treatment duration at baseline. Treatment duration was assigned positive values if therapy preceded the baseline and negative values if therapy was initiated post‐baseline within an allowed 60‐day window. The prognostic models in the pooled cohort were additionally adjusted for therapy category at baseline. We then compared these fully adjusted models to the models including the principal components and the adjustment variables only (without NfL), and prognostic models including NfL and the adjustment variables only (without the principal components). Proportionality of the hazards was evaluated using the global test and visual inspection of Schoenfeld's residuals. All predictors were scaled before modeling and all models are reported using hazard ratios (HR) and their 95% confidence intervals.

The baseline hazard functions for all predictive models were estimated with Nelson‐Aalen non‐parametric estimator [34]. Individual outcomes (cumulative hazards of relapse, CDW, CDI) were predicted using the coefficients obtained from the models described above. The discrimination ability (also commonly referred to as accuracy in medical literature) of the individual predictions was then compared using bootstrap validation with 5000 repetitions over a prediction horizon of 4 years and quantified with Uno's concordance index for each iteration and averaged for each outcome. Uno's concordance index is a measure of discrimination that assesses the concordance between the predicted and the observed cases without approximation to study‐specific censoring distribution for the cases that did not experience the event within the prediction horizon; instead, it utilises inverse probability weighting to account for censored cases [35, 36]. An Uno's c‐index that is greater than 50% means the proposed model is able to differentiate better than mere chance between the individuals at a higher and lower risk of experiencing the outcome event better than mere chance. Values of 0.8 or greater generally reflect good discrimination ability. Lastly, secondary analysis was carried out in a sub‐group treated with a single therapy for at least 6 months, who had not experienced a relapse within the 90 days preceding the baseline visit and in whom blood sample was collected within 10 days of the baseline visit. These more stringent criteria were applied to remove the potential confounding effects of recent relapses and therapeutic lag [37].

All analyses were performed using R software (version 4.0.2).

### 2.5 Ethics Statement

This study was approved by Melbourne Health Human Research and Ethics Committee (HREC/56529/MH‐2019) and was carried out in accordance with each site's approvals for the original studies and with written consent from all participants.

## 3 Results

### 3.1 Cohort Description

**FIGURE 1:**
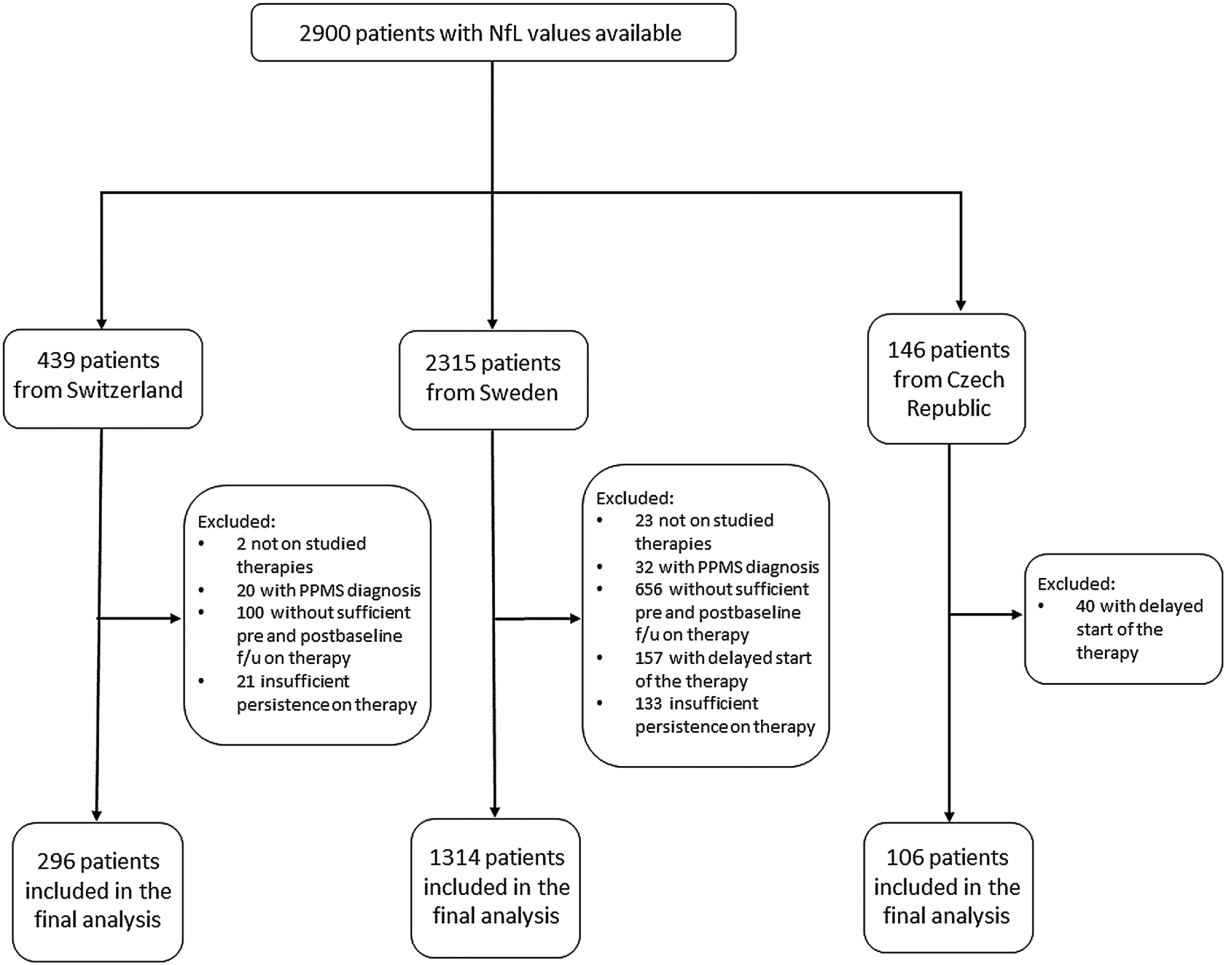
CONSORT diagram for case inclusion into the study.

1716 patients from the three study sites were eligible for the pooled analysis with an average 9 years of follow‐up (SD = 4). Three therapies – interferon β, fingolimod and natalizumab – were sufficiently represented with an adequate number of observed events for the DMT‐specific analysis (Figure 1, Table 1 and Table S1). Baseline characteristics were similar across the therapies except for MS duration and the time of the last relapse prior to the baseline. The shortest MS duration at the commencement of therapy was observed for interferons, while the patients utilising fingolimod had the longest interval to the most recent relapse activity (Table 1). Baseline characteristics of each site's cohort are shown in Table S1. Briefly, the eligible patients from the SET study were all treatment‐naïve, younger and with a shorter duration of MS. In contrast, patients from University Hospital of Basel were older and with a longer duration of MS. They were more likely to be treated more aggressively at the time of study inclusion and were mostly established on therapy. The cohort from Karolinska Institute showed a greater diversity to include patients with early MS as well as those with a more advanced disease, and overall, most patients had been treated at study inclusion for some time (Table S1).

**TABLE 1:** Baseline characteristics of the pooled cohort and the three sufficiently populated treatment groups.

	Interferon β	Fingolimod	Natalizumab	Pooled cohort
Included patients	554	307	369	1716
Follow‐up duration, years (mean ± SD)	9 ± 4	8 ± 3	10 ± 4	9 ± 4
Sex (female, %)	379 (69%)	198 (65%)	251 (68%)	1166 (68%)
Age, years (mean ± SD)	35 ± 11	41 ± 10	37 ± 10	38 ± 11
Age at MS onset, years	33 ± 10	30 ± 9	30 ± 9	32 ± 10
MS course (RRMS, *n*, %)	546 (99%)	302 (97%)	354 (96%)	1628 (95%)
MS duration, years (median, q1–q3)	1 (0–2)	9 (4–15)	5 (1–11)	3 (1–10)
EDSS (mean ± SD)	2.3 ± 1.8	2.1 ± 1.6	2.3 ± 1.7	2 (1–3)
ARR (median, q1–q3)	1 (0.4–1.1)	0.4 (0.2–0.7)	0.6 (0.3–1.2)	0.6 (0.2–1)
Patients previously treatment‐naive (*n*, %)	289 (52%)	13 (4%)	47 (13%)	472 (28%)
NfL concentration, *z* score (median, q1–q3)	1.6 (0.4–2.4)	0.9 (−0.2–1.8)	1.4 (0.5–2.2)	1.3 (0.1–2.1)
Treatment duration at baseline (days)	242 ± 657	291 ± 372	153 ± 397	200 ± 482
Time of the last relapse prior to the baseline (days) (median, q1–q3)	−135 (−377 – −44)	−619 (−1427 – −247)	−238 (−641 – −93)	−284 (−844 – −79)
NfL window^a^ (days) (median, q1–q3)	4 (1–35)	1 (0–14)	16 (1–37)	4 (1–28)

Abbreviations: ARR, Annualised relapse rate; DMT, Disease modifying therapy; EDSS, Expanded disability score scale; NfL, Neurofilament light chain; q1, First quartile; q3, Third quartile; SD, Standard deviation.

^a^ NfL window refers to the period of time between the study baseline when clinical assessment is performed and the blood sampling date.

The distribution of NfL *z*‐scores across different sites was in keeping with the clinical characteristics of each cohort (Table S1). Baseline characteristics of the excluded cases were similar to the baseline characteristics of the included participants (Table S2).

### 3.2 Contribution of NfL to Prognostics

**FIGURE 2:**
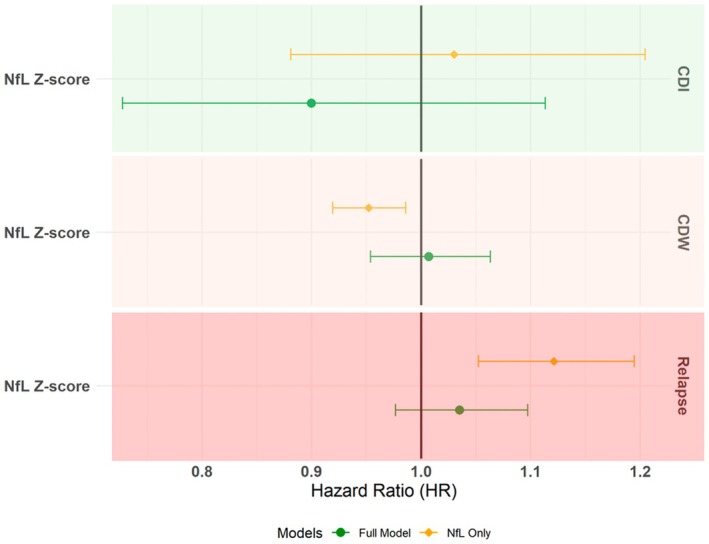
Forest plot for the contributions of patients' clinical and demographic characteristics to the prediction of NfL models of on‐treatment clinical outcomes with and without the background clinical information in the pooled cohort analysis.

We evaluated the overall prognostic value of NfL levels in the pooled cohort (Figure 2 and Figure S1). The full models, consisting of NfL levels, PC1‐3, treatment duration at baseline, and treatment category, found no evidence for an association of NfL levels with the three clinical outcomes, independent from the other modelled information. In line with our previous work, patients' clinical and demographic characteristics at baseline (represented by PC1 and PC2 with a minor contribution from phenotypic presentations at MS onset represented by PC3) were most consistently associated with the hazard of relapse, CDW, and CDI (Table S4) [15]. However, in the models without the PCs, a higher NfL level was associated with a higher probability of a relapse (Figure 2) (HR = 1.12, 95% CI: 1.05–1.19) and a lower probability of CDW (HR = 0.95, 95% CI: 0.92–0.99) (Table S4) (Figure 2).

The discrimination ability of the full models with the PCs was only marginally higher than the models without the PCs – with the most prominent increase in C for the model of CDI (Table 2). A sensitivity analysis included 366 patients who were treated with a single therapy for ≥ 6 months and who had not experienced a relapse within the 90 days preceding the baseline and had blood samples taken within 10 days of the baseline visit (Table S3). This analysis identified an association between higher NfL levels and higher probability of CDW, both when adjusted (HR = 1.19, 95% CI 1.09–1.29) and unadjusted for the PCs (HR = 1.21, 95% CI 1.1–1.32) (Table S5). The models with and without NfL showed similar C‐indices, whilst the models including NfL but not PCs showed lower C‐indices (Table S6).

**TABLE 2:** Discrimination ability of the models without/with NfL and without/with the principal components (PCs) for study outcomes assessed for the pooled cohort over 4 years post‐baseline.

	With PCs, without NfL	With PCs and NfL	Without PCs, with NfL only
Mean C‐index% for relapse (95% CI)	63.5 (63.4–63.5)	63.4 (63.4–63.5)	60.2 (60.1–60.2)
Mean C‐index% for CDW (95% CI)	56.4 (56.4–56.5)	56.4 (56.4–56.5)	55 (54.9–55.1)
Mean C‐index% for CDI (95% CI)	67.6 (67.5–67.7)	67.7 (67.6–67.8)	59.1 (59–59.2)

### 3.3 Contribution of NfL Levels to Prediction of Individual Treatment Response

Three DMTs were sufficiently represented with an adequate number of events to support the analysis of individual treatment response: interferon β (*n* = 554 patients), fingolimod (*n* = 307 patients), natalizumab (*n* = 369 patients) (Table 1).

**FIGURE 3:**
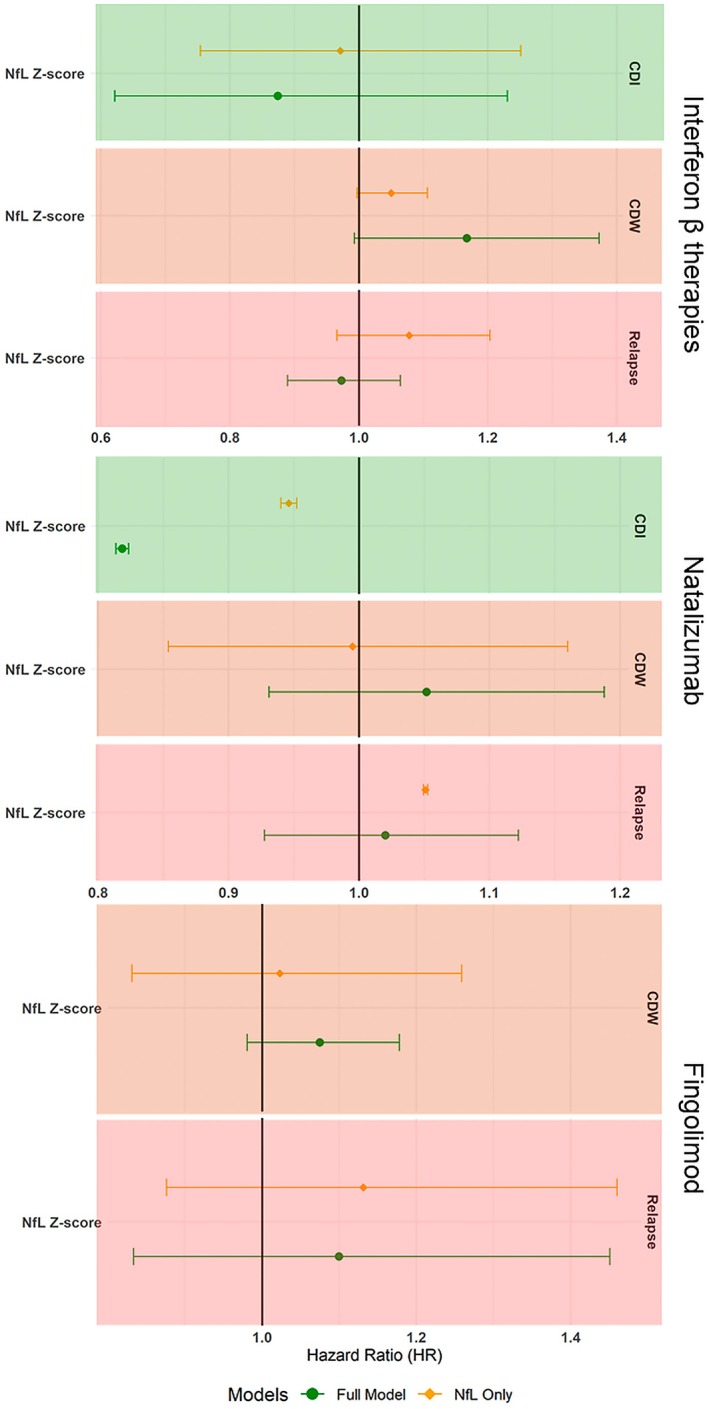
Forest plot for the contributions of NfL *z* scores to the prediction of on‐treatment clinical outcomes with and without PCs. The results are shown for adjusted multivariable Cox models for treatment‐specific models.

We did not find evidence for the association of NfL concentration with any of the outcomes in interferon β and fingolimod subgroups in either sets of the models (Figure 3 and Tables S7 and S8). In the natalizumab cohort, higher NfL was associated with a lower probability of CDI in the models with and without PCs (HR = 0.819, 95% CI: 0.814–0.823 and HR = 0.946, 95% CI: 0.94–0.952 respectively). In the absence of PCs, a weak association between higher NfL and higher risk of relapse was also observed (HR = 1.051, 95% CI: 1.049–1.053) (Figure 3 and Tables S7–S9).

Overall, the models with NfL showed similar discrimination ability as the models without NfL. Furthermore, models without PCs resulted in lower discrimination ability, highlighting the key role of the PCs representing patient characteristics in the prediction of the outcomes of treatment (Table 3).

**TABLE 3:** Discrimination ability of the models without/with NfL and without/with the principal components (PCs) for the study outcomes assessed for each of the treatment cohorts over 4 years post‐baseline.

	Interferon β			Fingolimod			Natalizumab		
	With PCs, without NfL	With PCs & NfL	Without PCs, with NfL only	Without NfL	With NfL	Without PCs, with NfL only	Without NfL	With NfL	Without PCs, with NfL only
Mean C‐index% for relapse (95% CI)	61.4 (61.4–61.5)	61.4 (61.4–61.5)	55.3 (55.2–55.4)	62.2 (62.1–62.3)	62.2 (62.1–62.2)	51.4 (51.3–51.5)	51.8 (51.7–51.9)	51.3 (51.2–51.4)	53.8 (53.7–53.9)
Mean C‐index% for CDW (95% CI)	58.6 (58.5–58.7)	60.3 (60.2–60.4)	52.9 (52.7–53)	54.9 (54.8–55.1)	54.3 (54.1–54.4)	46.2 (46.1–46.4)	55.8 (55.7–55.9)	56 (55.9–56.2)	51.5 (51.4–51.6)
Mean C‐index% for CDI (95% CI)	66.3 (66.1–66.4)	67.9 (67.7–68)	50.6 (50.5–50.8)	Models did not converge			50.6 (50.5–50.8)	65 (64.9–65.1)	57.6 (57.4–57.8)

## 4 Discussion

This study investigated the contribution of age normalised serum/plasma concentrations of NfL to a validated clinical predictive model [15] of relapses, disability worsening and disability improvement conditional on MS therapy. The results suggest that while in isolation NfL levels carried moderate prognostic value in the examined scenario, their added contribution to prognosis utilising comprehensive clinical and demographic information was not detectable. Thus, information such as patients' disability, prior relapses, prior use of DMTs and their effectiveness, age and MS course remain useful instruments accessible in routine practice to prognosticate medium‐term outcomes in individuals with MS.

The value of NfL levels as a stand‐alone prognostic biomarker of short‐ and long‐term outcomes in MS has been investigated in several cohorts [5, 6, 8, 9, 10, 11, 38]. NfL levels, adjusted for age and, in some instances, body mass index, have been shown to correlate with disease activity or disability outcomes over 2 years or longer. In a small cohort with high levels of NfL concentration, it was found that clinical MS activity may occur concurrently with increase in serum NfL; however, this study was not analytically adjusted for age [6]. This finding and other evidence [39] suggest that the window for detecting elevated NfL levels highlights its utility for finding latent disease activity; however, to what extent NfL elevation corresponds to disability needs further investigation.

Our results using real world NfL data under varying duration of treatment and proximity to recent relapse activity suggest that NfL levels helpe prognosticate relapses and disability outcomes, especially where detailed clinical and demographic information is unavailable. However, where patients' demographic and clinical status over time is thoroughly documented, their NfL level was not able to further improve the accuracy of predictions of treatment response. It is possible that the large number of influencing factors that may affect serum NfL concentrations play a role in its limited added prognostic contribution [3, 6].

Interestingly, in the pooled cohort analysis without adjustment for clinical and demographic characteristics (PCs), a higher concentration of NfL was associated with a lower probability of disability worsening. The sensitivity analysis among patients with 6‐month treatment persistence and freedom from relapses before the assessment of NfL found an association between higher NfL concentration and a higher hazard of disability worsening. This suggests the potential role for NfL as a prognostic marker among patients with seemingly well‐controlled disease on a stable therapy course. It also emphasises the importance of accounting for patient characteristics and the context of the NfL assessment (including stability on therapy) when evaluating its association with disability outcomes. Among patients who have recently commenced new therapy or have experienced a relapse that led to an increase in disability, elevated serum NfL may reflect the past neuronal damage due to the recent activity and may be less likely to be immediately followed by further deterioration in neurological function, especially if one views relapse as a treatable target [40]. On the contrary, among patients established on therapy and without clinically overt disease activity, elevated NfL levels may point to underlying neural tissue loss, which may be associated with disablity or focal episodic inflammation [41]. These results are in line with the finding of a recent study that investigated the baseline value of serum NfL with increased probability of confirmed disability worsening and becoming wheelchair‐bound [42] in people with primary or secondary progressive MS using clinical trial data. This study is notable in showing the effect of siponimod on reducing NfL levels, which is not linear throughout the study period and is more pronounced when contrast‐enhancing lesions are present on MRI [42].

In the context of the analyses within the three specific DMT groups, the adjustment for the baseline characteristics allows evaluation of the unique associations between NfL and the outcomes and its potential contribution to the discrimination ability of the predictive models. It is therefore interesting that the association of higher NfL concentration with lower probability of disability improvement during treatment with natalizumab is observed both with and without adjustment for clinical and demographic characteristics (Figure 3). One speculation is that the persistence of biomarkers of neuronal damage among patients treated with high‐efficacy therapies represents a poor prognostic marker and heralds a low probability of recovery from the presently accrued disability.

While the associations at the group level are found, the lack of increase in discrimination ability after addition of NfL may suggest that further studies following a more standardised sampling of NfL and focusing on specific clinical scenarios are required before NfL is sufficiently accurate to guide treatment choice in individuals [43] One step in this direction is represented by a study of reference NfL values normalised for age and body mass index, based on a large sample of healthy individuals and patients from the Swiss MS cohort [5].

The ability of NfL to predict significant impairment of gait (EDSS ≥ 6) over longer periods of time (8 or 15 years post‐baseline) was less reliable than short‐term prediction over 1 year of confirmed disability worsening [5, 9]. This most likely reflects the fact that NfL is a measure of recent neuronal breakdown and its underlying pathobiological processes are highly modifiable by therapy over the long term [5, 7, 12]. Furthermore, while being specific for neuronal damage, neuronal breakdown is the final step in a broad spectrum of processes (including normal ageing, neurotrauma, peripheral neuropathy, hyperglycaemia, etc.) [4] and therefore, NfL is not specific to MS. The practical motivation for our present study was to quantify the additive prognostic value of blood NfL as an add‐on to easily accessible predictors of individual treatment responses in an available multicentre setting. Clinicians routinely consider information such as prior and recent relapse frequency, on‐treatment breakthrough relapses, recovery from previous relapses, age, disease duration and clinical localisation of neurological disability in treatment decisions. We compared the ability to identify the individuals at risk of disease worsening between models enriched with this clinical background versus models with only NfL using Uno's concordance index. Concordance indices are widely used to quantify discrimination ability of the prediction models. Uno's iteration of the C index provides a metric which is robust to model misspecification and accounts for censored cases without assuming study‐specific censoring distribution [36]. The broad range of clinical prognostic information has been implemented in various prognostic tools, including age at MS onset [44, 45, 46], relapse activity in the preceding year [44, 47, 48], the presenting symptoms of MS or symptoms of prior relapses [44, 45, 49, 50]. However, the analytical frameworks limit the number of predictors that can be evaluated simultaneously. Especially where study cohorts' sizes are limited and the prognostic markers are collinear, comprehensive modelling may lead to underpowered or overfitted models. In these instances, researchers face the need to perform variable selection – ideally based on the detailed knowledge of their joint distributions and causal pathways. Methods such as stepwise selection of variables may lead to spurious results and are discouraged [51]. In designing the Crystal Ball model, we have circumvented the need to select variables by reducing the dimensionality of the information matrix using principal component analysis [17]. This allowed us to reduce the amount of multiplicate information and only retain features in patient history and disease phenotype that carry unique clinical value. This approach has performed well at reducing dimensionality of multivariable models in fields other than neurology [52]. Furthermore, we have previously illustrated the use of principal component analysis and the generalizability of the Crystal Ball model in predicting individual treatment response [15, 18, 30]. Implemented in the present study, it shows that comprehensive evaluation of clinical data enables an informative prediction of relapses, disability worsening and disability improvement at the group level. Most importantly, assessing the additive value of novel potential predictors in conjunction with the established prognostic models allows us to evaluate their value in the context of the currently accessible prognostics.

This study is subject to several limitations. We conducted this study by collating data from three MS cohorts with different data structures and different study protocols [16, 19]. To mitigate the effect of this heterogeneity, variables and their definitions were mapped across the three data sources by application of a harmonised data quality process [53]. Furthermore, these cohorts have been launched at different treatment epochs and may capture a broad range of treatment strategies. However, a broad range of represented treatment scenarios allows better generalisability of the prognostic models to MS populations globally. The analysis was either stratified by DMTs or adjusted for therapy (in the pooled cohort) and its duration to mitigate the effect of heterogeneity of DMT choice. The size of the eligible cohorts with NfL data enabled us to study individual prediction of treatment response in only three DMTs, albeit these are historically among the most used MS therapies. Moreover, we were unable to adjust NfL values for body mass index, given that this information was not available. On the other hand, we used normalised NfL values for age and adjusted the analyses for study site and laboratory platform as explained above [5, 29, 54]. NfL was measured in two different types of samples and with two different methods. NfL values in serum and plasma have been shown to have high correlation; however, despite the quality control and assurance protocols that have been followed, systematic differences between laboratory platforms might be present [55]. Finally, as this approach uses principal component analysis, extracting the exact contribution of individual characteristics (such as pre‐baseline relapse rate) towards the overall risk is not easily feasible.

## 5 Conclusion

In conclusion, this study showed that while serum/plasma NfL level carry a prognostic value, its value is strongly complemented by the clinicodemographic information and often masked in their presence. Furthermore, while the clinical markers help estimate future individual response to different MS therapies, NfL levels does not additionally predict treatment responders and non‐responders. NfL, as a marker of change underpinning overt or latent progression, seems to be most informative among patients with clinically and radiologically stable MS treated with established disease‐modifying therapy.

## Author Contributions

N.M., S.S., C.B.M. and T.K. contributed to study design, data collation, study analysis and manuscript preparation. J.K., E.K.H., D.H., J.H., T.O., M.B., T.J.K. and B.V.T. contributed to study design. J.K., J.H., T.O., M.B., T.J.K., D.L., P.B., E.K.H., D.H., P.K., T.U., A.M., K.B., B.V.T. and I.K. contributed to data collection and collation.

## Funding

This study was funded by a research grant from the National Health and Medical Research Council of Australia.

## Conflicts of Interest

E.K.H. has received honoraria/research support from Biogen, Merck Serono, Novartis, Roche and Teva and has served as a member of advisory boards for Actelion, Biogen, Celgene, Merck Serono, Novartis and Sanofi Genzyme. D.H. was supported by Cooperation Program in Neuroscience, Charles University; by the project National Institute for Neurological Research (Programme EXCELES, ID Project No. LX22NPO5107) – Funded by the European Union – Next Generation EU, and by General University Hospital in Prague project MH CZ‐RVO‐VFN64165. She also received compensation for travel, speaker honoraria, and consultant fees from Biogen, Novartis, Merck, Bayer, Sanofi Genzyme, Roche and Teva, as well as support for research activities from Biogen Idec. T.U. received financial support for conference travel and honoraria from Biogen, Novartis, Roche, Bristol Myers Squibb and Merck, as well as support for research activities from Biogen and Sanofi. He also received funding from the Czech Ministry of Health project (NU22‐04‐00193), the Charles University Cooperation Program in Neuroscience, and the National Institute for Neurological Research project funded by the European Union – Next Generation EU (Programme EXCELES, ID Project no. LX22NPO5107). T.O. has received advisory board/lecture honoraria as well as unrestricted MS research grants from Biogen, Merck, Novartis and Sanofi, none of which have any relation to the current paper. Academic MS research grants have been received from the Swedish Research Council, the Wallenberg Foundation, the Swedish Brain Foundation and Margaretha af Ugglas Foundation. J.H. declares research grants outside of this study from Biogen, Bristol‐Myers‐Squibb, Janssen, Merck KGaA, Novartis, Roche, and Sanofi‐Genzyme, and speaker's fees or fees for serving on advisory boards for Biogen, Bristol‐Myers‐Squibb, Janssen, Merck KGaA, Novartis, Sandoz, Sanofi‐Genzyme and Teva. I.K. has received lecture honoraria from Merck and a research grant from Pfizer. K.B. Katherine Buzzard has received speaker's fees or honoraria for serving on advisory boards for Biogen, Merck, Roche, Novartis, UCB, Alexion, Argenx, and CSL. T.K. served on scientific advisory boards or as a consultant for MS International Federation and World Health Organisation, Therapeutic Goods Administration, BMS, Roche, Janssen, Genzyme, Novartis, Merck and Biogen, received conference travel support and/or speaker honoraria from WebMD Global, Merck, Sandoz, Novartis, Biogen, Roche, Eisai, Genzyme, Teva and BioCSL, and received research or educational event support from Biogen, Novartis, Genzyme, Roche, Celgene and Merck. The other authors declare no conflicts of interest.

## Supporting information


**Table S1:** Baseline characteristics of the study cohort grouped by study site. ARR, annualised relapse rate; DMT, disease modifying therapy; EDSS, Expanded Disability Score Scale; NfL, Neurofilament light chain; q1, first quartile; q3, third quartile; SD, standard deviation.
**Table S2:** Baseline characteristics of the excluded cases.
**Table S3:** Baseline characteristics of the sensitivity analysis cohort per study site. ARR, annualised relapse rate; DMT, disease modifying therapy; EDSS, Expanded disability score scale; NfL, Neurofilament light chain; q1, first quartile; q3, third quartile; SD, standard deviation.
**Table S4:** Hazard ratios and their 95% confidence interval for multivariate Cox models in the pooled cohort. CI, Confidence Interval; HR, Hazard Ratio; ***p‐value < 0.001.
**Table S5:** Hazard ratios and their 95% confidence interval for multivariate Cox models in the sensitivity analysis cohort. Cox proportional hazards model for probability of disability improvement did not converge hence results are not shown. CI, Confidence Interval; HR, Hazard Ratio; mAb, monoclonal antibody therapy; ***p‐value < 0.001.
**Table S6:** Discrimination ability of the models without and with NfL for study outcomes assessed for the sensitivity analysis cohort over 4 years post‐baseline.
**Table S7:** Contributions of patients' clinical and demographic characteristics to the prediction of on‐treatment clinical outcomes with and without inclusion of serum/plasma NfL concentration. The results are shown for adjusted multivariable Cox models for treatment‐specific models. CI, Confidence Interval; HR, Hazard Ratio; PC, Principal Component; *p‐value < 0.05, **p‐value < 0.01, ***p‐value < 0.001.
**Table S8:** Contributions of the baseline NfL levels to the prediction of on‐treatment clinical outcomes as the sole predictor. The results are shown for Cox models adjusted for therapy duration for treatment‐specific models. CI, Confidence Interval; HR, Hazard Ratio.
**Table S9:** Frequencies of the observed relapse and disability worsening events and their overlap.
**Figure S1:** Distribution of NfL Z‐scores versus age per study site.

## Data Availability

Data used for this study was contributed by the collaborating sites; any access to the study data remains at the discretion of the respective data custodians.
