# Supplementary material for: Neurofilament Light Chain Concentration in the Prediction of Treatment Response in Multiple Sclerosis
Source: Eur J Neurol. 2026 Feb 2;33(2):e70505. doi: 10.1111/ene.70505 (PMC12862443; doi:10.1111/ene.70505)

|  | Karolinska Institute | University Hospital of Basel | Prague General Hospital | Total |
| --- | --- | --- | --- | --- |
| DMT (n,% per centre) |  |  |  |  |
| Alemtuzumab | 33 (2.5%) | 1 (0.3%) | - | 34 (2%) |
| Interferon beta-1 a, IM | 233 (17.5%) | 11 (3.7%) | 106 (100%) | 350 (20.4%) |
| Interferon beta-1 b | 87 (6.6%) | 12 (4.1%) | - | 99 (5.8%) |
| Interferon beta-1 a, SC | 93 (7.1) | 4 (1.4%) | - | 97 (5.7%) |
| Peginterferon | 6 (0.5%) | 2 (0.7%) | - | 8 (0.5%) |
| Glatiramer acetate | 61 (4.6%) | 13 (4.4%) | - | 74 (4.3%) |
| Dimethyl fumarate | 195 (14.8%) | 37 (12.5%) | - | 232 (13.5%) |
| Fingolimod | 152 (11.6%) | 155 (52.4%) | - | 307 (17.9%) |
| Natalizumab | 345 (26.3%) | 24 (8.1%) | - | 369 (21.5%) |
| Ocrelizumab | - | 19 (6.4%) | - | 19 (1.1%) |
| Rituximab | 57 (4.3%) | 11 (3.7%) | - | 68 (4%) |
| Teriflunomide | 52 (4%) | 7 (2.4%) | - | 59 (3.4%) |
| Follow-up duration (mean ± sd) | 9 ± 4 | 6 ±2 | 12 ± 2 | 9 ± 4 |
| Sex (female, %) | 898 (68%) | 204 (69%) | 64 (60%) | 1166 (68%) |
| Age, years (mean±sd) | 37 ± 10 | 43 ± 11 | 30 ± 8 | 38 ± 11 |
| Age at MS onset, years | 32 ± 10 | 32 ± 10 | 30 ± 8 | 32 ± 10 |
| MS course (RRMS,n,%) | 1279 (97%) | 243 (82%) | 106 (100%) | 1628 (95%) |
| MS duration, years (median, q1-q3) | 3 (1-9) | 10 (4-17) | 0 | 3 (1-10) |
| EDSS (median, q1-q3) | 2 (1-3) | 2 (1.5-3) | 2.5 (1.5-4) | 2 (1-3) |
| ARR (median, q1-q3) | 0.7 (0.2-1.1) | 0.4 (0.2-0.7) | 1 | 0.6 (0.2-1) |
| Patients previously treatment-naive (n,%) | 366 (28%) | 0 | 106 (100%) | 472 (28%) |
| NfL concentration, z score (median, q1-q3) | 1.5 (0.4-2.2) | 0.2 (-0.7 - 1.1) | 1.8 (0.4-2.8) | 1.3 (0.1-2.1) |
| Treatment duration at baseline (days)(median, q1-q3) | 109 ± 277 | 690 ± 841 | -49 ± 7.1 | 200 ± 482 |
| Time of the last relapse prior to the baseline (days) (median, q1-q3) | -245 (-767- -84) | -706 (-1530 - -423) | -26 (-50 - -11) | -284 (-844 – -79) |
| NfL window (days) (median, q1-q3) | 12 (1-36) | 0 | 0 | 4 (1-28) |

Table S1: Baseline characteristics of the study cohort grouped by study site. DMT: disease modifying therapy, sd: standard deviation, q1: first quartile, q3: third quartile, EDSS: Expanded Disability Score Scale, ARR: annualized relapse rate, NfL: Neurofilament light chain

Table S2: Baseline characteristics of the excluded cases

|  | Karolinska Institute | University Hospital of Basel | Prague General Hospital |
| --- | --- | --- | --- |
| Excluded participants | 1001 | 143 | 40 |
| Age, years (mean, sd) | 38 ± 11 | 44 ± 12 | 29 ± 8 |
| Age at onset, years (mean, sd) | 33 ± 10 | 34 ± 12 | 29 ± 8 |
| Follow up, years (mean, sd) | 8 ± 4 | 8 ± 2 | 12 ± 1 |
| Sex (,%) | 716 (72%) | 83 (59%) | 32 (80%) |
| MS duration, years (median, q1-q3) | 3 (1-7) | 7 (3-15) | 0 |
| EDSS (median, q1-q3) | 2 (1-2.5) | 1.5 (1-3) | 2.25 (1.5-4) |

Table S3: Baseline characteristics of the sensitivity analysis cohort per study site. DMT: disease modifying therapy, sd: standard deviation, q1: first quartile, q3: third quartile, EDSS: Expanded disability score scale, ARR: annualized relapse rate, NfL: Neurofilament light chain

|  | Sensitivity analysis | | |
| --- | --- | --- | --- |
|  | Karolinska Institute | Basel University Hospital | Total |
| Included patients | 125 | 241 | 366 |
| Follow-up duration (mean ± sd) | 10 ± 5 | 6 ± 2 | 7 ± 4 |
| Sex (female, %) | 92 (74%) | 161 (67%) | 253 (69%) |
| Age, years (mean±sd) | 38 ± 10 | 43 ± 11 | 41 ± 11 |
| Age at MS onset, years (mean±sd) | 33 ± 9 | 32 ± 10 | 32 ± 10 |
| MS course (RRMS, n, %) | 124 (99%) | 194 (81%) | 318 (87%) |
| MS duration, years (median, q1-q3) | 3 (2–7) | 10 (4–16) | 7 (2–13) |
| EDSS (median, q1-q3) | 1.5 (1–2.5) | 2.5 (1.5–3.5) | 2 (1.125–3) |
| ARR (median, q1-q3) | 0.7 (0.3–1.1) | 0.4 (0.2–0.7) | 0.5 (0.2–0.8) |
| NfL concentration, z score (median, q1-q3) | 1.4 (0.1 – 2.4) | 0.2 (-0.7 – 1) | 0.5 (-0.5 – 1.5) |
| Treatment duration at baseline,(days) (median, q1-q3) | 401 (275–742) | 546 (351–777) | 506 (336–775) |

| Pooled cohort | | | |
| --- | --- | --- | --- |
| Relapse | | | |
|  | Without NfL | With NfL | With NfL, without PCs |
| Observed events, n (%) | 474 (28%) | | |
| Time to relapse (days) (median, q1-q3) | 403 (182-803) | | |
| NfL, HR (95%CI) | - | 1.04 (0.98-1.1) | 1.12 (1.05-1.19)*** |
| PC1, HR (95%CI) | 2.85 (1.34-6.08)** | 2.78 (1.33-5.81)** | - |
| PC2, HR (95%CI) | 3.21 (1.81-5.66)*** | 3.1 (1.81-5.32)*** | - |
| PC3, HR (95%CI) | 0.89 (0.64-1.23) | 0.89 (0.65-1.22) | - |
| Confirmed Disability Worsening | | | |
|  | Without NfL | With NfL | Without PCs |
| Observed events, n (%) | 193 (11%) | | |
| Time to CDW (days) (median, q1-q3) | 730 (374-1188) | | |
| NfL, HR (95%CI) | - | 1.01 (0.95-1.06) | 0.95 (0.92-0.99)** |
| PC1, HR (95%CI) | 0.43 (0.26-0.7)*** | 0.43 (0.25-0.73)** | - |
| PC2, HR (95%CI) | 0.44 (0.27-0.71)*** | 0.44 (0.25-0.75)** | - |
| PC3, HR (95%CI) | 0.82 (0.8-0.85)*** | 0.82 (0.8-0.85)*** | - |
| Confirmed Disability Improvement | | | |
|  | Without NfL | With NfL | Without PCs |
| Observed events, n (%) | 139 (8%) | | |
| Time to CDI (days) (median, q1-q3) | 366 (204-759) | | |
| NfL, HR (95%CI) | - | 0.9 (0.73-1.11) | 1.03 (0.88-1.2) |
| PC1, HR (95%CI) | 0.6 (0.07-5.05) | 0.63 (0.08-4.81) | - |
| PC2, HR (95%CI) | 1.4 (0.34-5.74) | 1.53 (0.43-5.47) | - |
| PC3, HR (95%CI) | 0.37 (0.15-0.97)* | 0.36 (0.14-0.98)* | - |

Table S4: Hazard ratios and their 95% confidence interval for multivariate Cox models in the pooled cohort. HR: Hazard Ratio, CI: Confidence Interval, ***P-value<0.001

Table S5: Hazard ratios and their 95% confidence interval for multivariate Cox models in the sensitivity analysis cohort. Cox proportional hazards model for probability of disability improvement did not converge hence results are not shown. HR: Hazard Ratio, CI: Confidence Interval, mAb: monoclonal antibody therapy; ***P-value<0.001

|  | **Relapse** | | | **Confirmed Disability Worsening** | | |
| --- | --- | --- | --- | --- | --- | --- |
|  | **Without NfL** | **with NfL** | **Without PCs** | **Without NfL** | **with NfL** | **Without PCs** |
| **Observed events, n (%)** | 104 (28%) | | | 50 (14%) | | |
| **NfL,**  **HR (95% CI)** | - | 1.05 (0.86-1.27) | 1.12 (0.9-1.38) | - | 1.19 (1.09-1.29)*** | 1.21 (1.1-1.32)*** |
| **PC1,**  **HR (95% CI)** | 50.01 (17.7-141.29)*** | 46.82 (22.18-98.82)*** | - | 0.28 (0.27-0.29)*** | 0.24 (0.22-0.27)*** | - |
| **PC2,**  **HR (95% CI)** | 52.64 (16.94-163.57)*** | 49.13 (21.19-113.86)*** | - | 0.31 (0.28-0.36)*** | 0.27 (0.2-0.37)*** | - |
| **PC3,**  **HR (95% CI)** | 1.23 (1.23-1.24)*** | 1.23 (1.22-1.25) | - | 0.6 (0.54-0.67)*** | 0.61 (0.54-0.7)*** | - |

Table S6: Discrimination ability of the models without and with NfL for study outcomes assessed for the sensitivity analysis cohort over 4 years post-baseline

|  | Without NfL | With NfL | Without PCs |
| --- | --- | --- | --- |
| Mean C-index% for Relapse (95% CI) | 65.6 (65.5-65.7) | 65.3 (65.2-65.3) | 56.7 (56.6-56.7) |
| Mean C-index% for CDW (95% CI) | 67.1 (67-67.2) | 67.3 (67.1-67.4) | 56.7 (56.6-56.8) |

|  | Interferon β | | Fingolimod | | Natalizumab | |
| --- | --- | --- | --- | --- | --- | --- |
| Relapse | | | | | | |
|  | Without NfL | With NfL | Without NfL | With NfL | Without NfL | With NfL |
| observed events, n (%) | 237 (43%) | | 80 (26%) | | 67 (18%) | |
| NfL, HR (95%CI) | - | 0.97 (0.89-1.06) | - | 1.1 (0.83-1.45) | - | 1.02 (0.93-1.12) |
| PC1, HR (95%CI) | 1.15 (1.06-1.25)*** | 1.16 (1.06-1.26)*** | 11.04 (7.67-15.88)*** | 9.29 (9.16-9.42)*** | 1.72 (0.25-11.68) | 1.67 (0.21-13.43) |
| PC2, HR (95%CI) | 1.74 (1.49-2.02)*** | 1.76 (1.58-1.97)*** | 9.68 (6.16-15.2)*** | 8.12 (7.49-8.8)*** | 1.68 (0.3-9.52) | 1.62 (0.24-11) |
| PC3, HR (95%CI) | 0.64 (0.56-0.74)*** | 0.64 (0.57-0.71)*** | 1.29 (1.25-1.34)*** | 1.31 (1.22-1.41)*** | 0.89 (0.81-0.98)* | 0.89 (0.82-0.97)** |
| Confirmed Disability Worsening | | | | | | |
|  | Without NfL | With NfL | Without NfL | With NfL | Without NfL | With NfL |
| Observed events, n (%) | 56 (10%) | | 41 (13%) | | 44 (12%) | |
| NfL, HR (95%CI) | - | 1.17 (0.99-1.37) | - | 1.08 (0.98-1.18) | - | 1.05 (0.93-1.19) |
| PC1, HR (95%CI) | 0.78 (0.1-5.79) | 0.74 (0.1-5.59) | 0.4 (0.27-0.61)*** | 0.35 (0.22-0.55)*** | 0.79 (0.49-1.25) | 0.71 (0.54-0.94)* |
| PC2, HR (95%CI) | 0.65 (0.13-3.3) | 0.58 (0.1-3.29) | 0.32 (0.27-0.39)*** | 0.27 (0.22-0.34)*** | 0.65 (0.37-1.14) | 0.59 (0.41-0.85)** |
| PC3, HR (95%CI) | 1.47 (0.72-2.97) | 1.52 (0.8-2.88) | 0.98 (0.62-1.54) | 0.99 (0.64-1.53) | 1.11 (1.09-1.13)*** | 1.11 (1.1-1.13)*** |
| Confirmed Disability Improvement | | | | | | |
|  | Without NfL | With NfL | Without NfL | With NfL | Without NfL | With NfL |
| Observed events, n (%) | 39 (7%) | | 17 (6%) | | 47 (13%) | |
| NfL, HR (95%CI) | - | 0.87 (0.62-1.23) | Models did not converge | | - | 0.82 (0.81-0.82)*** |
| PC1, HR (95%CI) | 2.68 (0.53-13.61) | 2.55 (0.42-15.34) |  |  | 5.34 (2.54-11.21)*** | 8.13 (4-16.5)*** |
| PC2, HR (95%CI) | 3 (0.85-10.63) | 3.07 (0.89-10.53) |  |  | 4.23 (2.69-6.65)*** | 6.44 (4.2-9.89)*** |
| PC3, HR (95%CI) | 0.72 (0.34-1.53) | 0.68 (0.27-1.71) |  |  | 0.65 (0.53-0.8)*** | 0.64 (0.52-0.77)*** |

Table S7: Contributions of patients’ clinical and demographic characteristics to the prediction of on-treatment clinical outcomes with and without inclusion of serum/plasma NfL concentration. The results are shown for adjusted multivariable Cox models for treatment-specific models. PC: Principal Component, HR: Hazard Ratio, CI: Confidence Interval *P-value< 0.05, **P-value< 0.01, ***P-value<0.001

Table S8: Contributions of the baseline NfL levels to the prediction of on-treatment clinical outcomes as the sole predictor. The results are shown for Cox models adjusted for therapy duration for treatment-specific models. HR: Hazard Ratio, CI: Confidence Interval

| Models without the principal components | | | |
| --- | --- | --- | --- |
|  | **Interferon β** | **Fingolimod** | **Natalizumab** |
| Relapse | | | |
| Observed events, n (%) | 237 (43%) | 80 (26%) | 67 (18%) |
| NfL, HR (95%CI) | 1.08 (0.97-1.2) | 1.13 (0.88-1.46) | 1.051 (1.049-1.053)*** |
| Confirmed Disability Worsening | | | |
| Observed events, n (%) | 56 (10%) | 41 (13%) | 44 (12%) |
| NfL, HR (95%CI) | 1.1 (0.99-1.11) | 1.02 (0.83-1.26) | 1 (0.85-1.16) |
| Confirmed Disability Improvement | | | |
| Observed events, n (%) | 39 (7%) | 17 (6%) | 47 (13%) |
| NfL, HR (95%CI) | 0.97 (0.75-1.25) | Models did not converge | 0.95 (0.94-0.95)*** |

Table S9: Frequencies of the observed relapse and disability worsening events and their overlap

|  | **Interferon β** | | |
| --- | --- | --- | --- |
|  |  | **Confirmed Disability Worsening** | |
|  |  | yes | no |
| **Relapse** | yes | 32 | 205 |
|  | no | 24 | 293 |
|  | **Fingolimod** | | |
|  |  | **Confirmed Disability Worsening** | |
|  |  | yes | no |
| **Relapse** | yes | 18 | 62 |
|  | no | 23 | 204 |
|  | **Natalizumab** | | |
|  |  | **Confirmed Disability Worsening** | |
|  |  | yes | no |
| **Relapse** | yes | 17 | 50 |
|  | no | 27 | 275 |
|  | **Pooled cohort** | | |
|  |  | **Confirmed Disability Worsening** | |
|  |  | yes | no |
| **Relapse** | yes | 81 | 393 |
|  | no | 112 | 1130 |

Figures S1: Distribution of NfL Z-scores vs age per study site


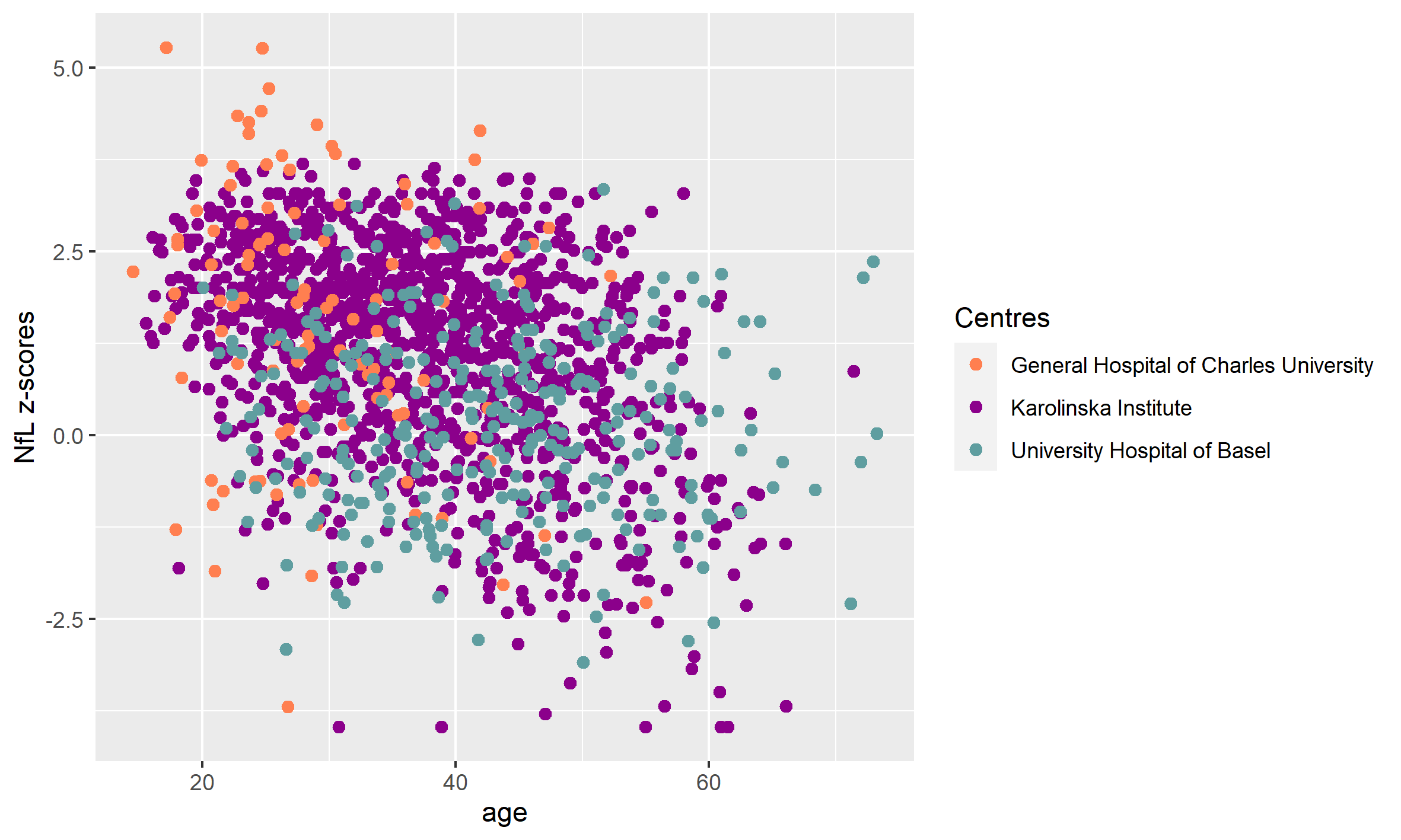

Supplement: Supplementary file 1 — Table S1: Baseline characteristics of the study cohort grouped by study site. ARR, annualised relapse rate; DMT, disease modifying therapy; EDSS, Expanded Disability Score Scale; NfL, Neurofilament light chain; q1, first quartile; q3, third quartile; SD, standard deviation. Table S2: Baseline characteristics of the excluded cases. Table S3: Baseline characteristics of the sensitivity analysis cohort per study site. ARR, annualised relapse rate; DMT, disease modifying therapy; EDSS, Expanded disability score scale; NfL, Neurofilament light chain; q1, first quartile; q3, third quartile; SD, standard deviation. Table S4: Hazard ratios and their 95% confidence interval for multivariate Cox models in the pooled cohort. CI, Confidence Interval; HR, Hazard Ratio; ***p‐value < 0.001. Table S5: Hazard ratios and their 95% confidence interval for multivariate Cox models in the sensitivity analysis cohort. Cox proportional hazards model for probability of disability improvement did not converge hence results are not shown. CI, Confidence Interval; HR, Hazard Ratio; mAb, monoclonal antibody therapy; ***p‐value < 0.001. Table S6: Discrimination ability of the models without and with NfL for study outcomes assessed for the sensitivity analysis cohort over 4 years post‐baseline. Table S7: Contributions of patients' clinical and demographic characteristics to the prediction of on‐treatment clinical outcomes with and without inclusion of serum/plasma NfL concentration. The results are shown for adjusted multivariable Cox models for treatment‐specific models. CI, Confidence Interval; HR, Hazard Ratio; PC, Principal Component; *p‐value < 0.05, **p‐value < 0.01, ***p‐value < 0.001. Table S8: Contributions of the baseline NfL levels to the prediction of on‐treatment clinical outcomes as the sole predictor. The results are shown for Cox models adjusted for therapy duration for treatment‐specific models. CI, Confidence Interval; HR, Hazard Ratio. Table S9: Frequencies of the observed rela [file ENE-33-e70505-s001.docx]
